# Proteomic Analysis of Iodinated Contrast Agent-Induced Perturbation of Thyroid Iodide Uptake

**DOI:** 10.3390/jcm9020329

**Published:** 2020-01-23

**Authors:** Maha Hichri, Georges Vassaux, Jean-Marie Guigonis, Thierry Juhel, Fanny Graslin, Julien Guglielmi, Thierry Pourcher, Béatrice Cambien

**Affiliations:** 1 Université Côte d’Azur, UMR E4320, CEA, F-06107 Nice, France; hichri.maha@gmail.com (M.H.); vassaux@ipmc.cnrs.fr (G.V.); Jean-Marie.Guigonis@unice.fr (J.-M.G.); fanny.graslin@unice.fr (F.G.); Julien.GUGLIELMI@unice.fr (J.G.); Thierry.POURCHER@unice.fr (T.P.); 2 Université Côte d’Azur, INSERM, CNRS, IPMC, F-06560 Valbonne, France; 3 Plateforme “Bernard Rossi”, Faculté de médecine, Université Côte d’Azur, F-06107 Nice, France; 4 INSERM, Institute for Research on Cancer and Aging, Université Côte d’Azur, F-06107 Nice, France; Thierry.JUHEL@unice.fr; 5 Université Côte d’Azur, INSERM, UMR E4320, CEA, F-06107 Nice, France

**Keywords:** iodinated contrast media, free iodide, thyroid, microSPECT/CT imaging, proteomics

## Abstract

(1) Background: We recently showed that iodinated contrast media (ICM) reduced thyroid uptake of iodide independently of free iodide through a mechanism different from that of NaI and involving a dramatic and long-lasting decrease in Na/I symporter expression. The present study aimed at comparing the response of the thyroid to ICM and NaI using a quantitative proteomic approach. (2) Methods: Scintiscans were performed on ICM-treated patients. Micro Single-Photon Emission Computed Tomography (microSPECT/CT) imaging was used to assess thyroid uptakes in ICM- or NaI-treated mice and their response to recombinant human thyroid-stimulating hormone. Total thyroid iodide content and proteome was determined in control, NaI-, or ICM-treated animals. (3) Results: The inhibitory effect of ICM in patients was selectively observed on thyroids but not on salivary glands for up to two months after a systemic administration. An elevated level of iodide was observed in thyroids from NaI-treated mice but not in those from ICM animals. Exposure of the thyroid to NaI modulates 15 cellular pathways, most of which are also affected by ICM treatment (including the elF4 and P706SK cell signaling pathway and INSR identified as an upstream activator in both treatments). In addition, ICM modulates 16 distinct pathways and failed to affect thyroid iodide content. Finally, administration of ICM reduces thyroid-stimulating hormone (TSH) receptor expression which results in a loss of TSH-induced iodide uptake by the thyroid. (4) Conclusions: Common intracellular mechanisms are involved in the ICM- and NaI-induced reduction of iodide uptake. However, ICM fails to affect thyroid iodide content which suggests that the modulation of these common pathways is triggered by separate effectors. ICM also modulates numerous distinct pathways which may account for its long-lasting effect on thyroid uptake. These observations may have implications in the management of patients affected by differentiated thyroid carcinomas who have been exposed to ICM. They also provide the basis for the utilization of ICM-based compounds in radioprotection of the thyroid.

## 1. Introduction

In the thyroid, iodide is taken up from the blood plasma by follicular cells. This uptake is mediated by the Na/I symporter (NIS) protein present at the basolateral membrane of these cells [1]. Free iodide is oxidized outside the cells at the apical membrane with thyroid peroxidase enzyme (TPO), then it is covalently bound to tyrosine residues of the thyroglobulin protein once inside. The administration of high amounts of free iodide prevents entry of iodide into the thyroid. Mechanistically, this effect is the result of a physiological response, referred to as the Wolff–Chaikoff effect, in which the expression of the NIS protein is reduced [2].

Iodinated contrast media (ICM) are routinely administered intravenously to patients worldwide. ICM are not metabolized, show an exclusively extracellular distribution, are cleared by glomerular filtration, and eliminated by renal excretion (up to 90% within the first 24 h) [3]. A well-documented side effect of ICM administration is to dramatically decrease iodide or the iodide surrogate pertechnetate [4,5] uptake by the thyroid in humans [6,7,8,9,10,11,12,13]. This phenomenon has also been observed in rats [14], cats [15], and mice [16]. In humans, intravenous administration of ICM is known to impair thyroid uptake for up to eight weeks. This phenomenon compromises diagnostic thyroid scintigraphy and radio-iodine treatment of thyroid malignancies [6,7,8,9,10]. The latest guidelines recommend delaying radioactive iodide treatment in patients who have been exposed to ICM [9,11,12,13]. Historically, this reduced iodide uptake by thyroid tissues in response to ICM was attributed to the high amounts of free iodide associated with [17] and/or released from [9,18] the ICM formulation. Consistent with this hypothesis, urinary iodide content is elevated for four-to-six weeks before a return to normal levels [19,20,21,22]. Urinary iodide content has been used as a surrogate marker of the restoration of normal iodide uptake by thyroid tissues [12].

However, the involvement of free iodide in the reduction of iodide uptake by the thyroid in response to ICM has never been formally demonstrated. Recently, we showed, using molecular imaging in mice, that ICM reduces iodide uptake by the thyroid to a greater and longer-lasting degree than the free iodide found in ICM would explain [16]. In contrast, neither ICM nor its contaminating free iodide are able to directly compromise thyrocyte uptake in vitro [16]. In addition, the effect of ICM is specific to the thyroid as it does not affect the uptake of iodide by another NIS expressing tissue, the salivary glands, while administration of potassium iodide (KI) affects both organs. This selectivity of action of ICM to the thyroid was also evidenced in patients [16]. Here, we document that this uptake capacity in some patients remains low even two months after a single administration of ICM. Although indirect, these imaging data strongly suggest that, at the organ level, ICM inhibits thyroid iodide uptake through a mechanism different from that of KI. From a clinical point of view, this evidence casts doubt on the relevance and usefulness of the standard measurement of urinary iodide concentration to evaluate the delay between ICM administration and radioiodine therapy of patients with differentiated thyroid carcinoma. In addition, although rare, side effects of the use of ICM, such as thyrotoxicosis, hyperthyroidism, and hypothyroidism, have been reported, both in populations at risk and in some cases in individuals without previous thyroid dysfunction [9,23,24]. However, the main side effect observed in patients is the perturbation of iodide uptake by the thyroid. In this context, whether ICM induce intracellular events in the thyroid, identical to those triggered by free iodide (referred to as the Wolff–Chaikoff effect), remains to be determined.

As previous imaging data from our group provided indirect evidence that ICM inhibit thyroid iodide uptake through a mechanism different from that of NaI [16], the present study was precisely designed to provide strong additional molecular evidence of distinct intracellular events triggered by ICM and NaI in the thyroid using unbiased approaches. To get a general and comparative overview of ICM and NaI effects on thyrocytes protein expression, we applied the so-called TMT quantitative proteomics approach to thyroids from mice that had been treated with ICM or NaI. As a major advance for global protein quantification based on the introduction of isobaric mass tags in the samples, this approach simultaneously enables the global, accurate, and sensitive quantification of proteins across multiple samples [25,26].

In addition, we explored the effect of ICM on thyroid iodide content as well as on TSH-induced iodide uptake by the thyroid. Together, our data shed some new light on the mechanisms of thyroid inhibition induced by ICM.

## 2. Materials and Methods

### 2.1. Thyroid Uptake of ^99m^TcO_4_^−^ in Humans

An ICM-naïve patient and a patient who had received an intravenous injection of Iomeron 350 at two weeks and two months before scintigraphy were chosen for study. On the day of the scintigraphy, the patients were injected intravenously with a 1 MBq/kg activity and 600-s scans were obtained 15 min later. Scintiscans where the pinhole collimator is centered on the thyroid are shown. The Institutional Review Board approved this retrospective study, and the anonymized scans were obtained as part of routine medical examinations with consent of the patients.

### 2.2. Animals

Eight-week-old female C57Bl/6J mice were obtained from Janvier (Le Genest Saint Isle, France). Animal housing and procedures were conducted according to French Agriculture Ministry guidelines and were approved by the local ethics committee (Ciepal NCE/2014-211). According to the human dose of 0.6 g iodide/kg and to previous studies in mice [16], intravenous administrations of ICM were performed by injecting 100 µL Iomeron diluted 50/50 (vol/vol) with phosphate-buffered saline (corresponding to 18 mg iomeprol). Administration of NaI was performed by an intraperitoneal injection of 150 µL of a solution of 0.5% NaI, as previously described [16]. TSH treatment was performed by an intraperitoneal administration of 3.2 μg/mouse of thyrogen (Genzyme, Saint Germain en Laye, France) according to a validated protocol [5,27]. Thyroids were collected either 24 (Na I) or 48 h (ICM) after injection and were stored at −80 °C until protein or metabolite extraction.

### 2.3. Small-Animal MicroSPECT/CT Scans

^99m^Tc pertechnetate (^99m^TcO_4_^−^) was obtained from a freshly eluted ^99^Mo/^99m^Tc generator. Animals were administered intraperitoneally with activities of 20 MBq ^99m^TcO_4_^−^. Tracer uptake was measured one hour later using a dedicated microSPECT/CT scanner (eXplore speCZT CT120, GE, Boston, MA, USA) as previously described [28]. Reconstructed images were analyzed and quantified using AMIDE software [29]. Tri-dimensional regions of interest were drawn manually around the thyroid and salivary glands, as previously detailed [5,30]. Uptakes were expressed as percentages of the injected activity after decay correction [28].

### 2.4. Metabolites Extraction and Iodide Quantification

Metabolites from the thyroid were extracted in Methanol LC-MS grade (Millipore, Molsheim, France) and left overnight at −20 °C. The extract was centrifuged at 13,000× *g* for 15 min and the supernatant was dried with a rotary vacuum concentrator, and then dissolved in 30 µL 0.1% formic acid and 5% acetonitrile prior to mass spectrometric analysis. The analysis of all samples was performed using a DIONEX Ultimate 3000 HPLC system. Chromatographic analysis was performed using the following conditions: column: Phenomenex Synergi4 u Hydro-RP 80A 250 × 3.0 mm; column temperature: 40 °C; mobile phase A: 0.1% formic acid in water and mobile phase B: 0.1% formic acid in acetonitrile; flow rate: 0.9 mL/min. The LC gradient began with 5% B for 5 min and was ramped up to 95% B over 22 min. The column was re-equilibrated with 100% A for 3 min before the next run. MS analysis was carried out on a Thermo Scientific Exactive Plus benchtop Orbitrap mass spectrometer. The source conditions were as follows: ion source: heated electrospray ionization source (HESI II); ion source polarity: positive and negative ion mode; spray voltage: 3800 V in positive mode\2500 V in negative mode; vaporizer temperature: 350 °C; ion sweep gas: 1.0 units; ion transfer tube temperature: 300 °C; sheath gas pressure (N2): 60 units; auxiliary gas pressure (N2): 15 units. With the Exactive plus benchtop orbitrap mass spectrometer, generic conditions and an external mass calibration were used. The instrument was operated in full scan mode from *m/z* 67–1000. High-resolution accurate mass (HRAM) full-scan MS and top 5 MS/MS spectra were collected in a data-dependent fashion at a resolving power of 70,000 and 35,000 at FWHM *m/z* 200, respectively. The Stepped NCE (normalized collision energy) setting was 40. MS data were analyzed with MZMine 2.20 and were compared to a human database. Only Iodide metabolite was retained from an identified metabolite.

### 2.5. Protein Extraction

Proteins from the thyroid were extracted in RIPA buffer (NaCl 150 mM, EDTA 1 mM, Triton X-100 1%, SDS 0.1%, Tris-HCl pH7.5 10 mM) in the presence of protease and phosphatase inhibitors (Roche, Mannheim, Germany). The lysate was centrifuged at 14,000× *g* for 15 min, and the supernatant was quantified with the BCA Protein Assay Kit (Bio-Rad, Marnes-La-Coquette, France).

### 2.6. Tandem Mass Tag (TMT) Labelling

Heat-denatured protein samples (100 μg) were separated by SDS-PAGE. When marker dye reached 1 cm from the bottom of the gel, migration was stopped. Protein bands were excised from gel. Each sample was washed three times with 100 mM ammonium bicarbonate, dehydrated with acetonitrile, reduced with 10 mM dithiothréitol, (DTT), and alkylated using 55 mM Iodoacetamide. Samples were washed twice by 100 Mm Ammonium-bicarbonate and dried in a rotary vacuum concentrator. The dry gel spots were rehydrated with a 100 mM ammonium-bicarbonate buffer containing 40 ng/µL sequencing grade porcine trypsin (Promega, Madison, WI, USA) and digestion carried out at 37 °C for 16 h. The supernatant was removed and the tryptic peptides were extracted and dried in the rotary vacuum concentrator. Each of the TMT10plex reagents (0.8 mg, Thermo Fisher Scientific, Rockford, IL, USA) was resuspended in 41 μL of anhydrous acetonitrile and added to 100 μg of thyroid dried proteins digest dissolved in 100 μL of 50 mM Triethylammonium bicarbonate (TEAB) buffer. Control samples were labelled with 126, 127N, and 127C; Thyroids from NaI- and ICM-treated animals were labelled with 128N, 128C, 129N and 129C, 130N, 130C, respectively. After 1 h incubation at room temperature, the reaction was quenched by adding 8 μL of 5% hydroxylamine. The different TMT-labelled samples were cleaned-up using C18Spin Column (Thermo Fischer Scientific, Illkirch, France) then pooled at a 1:1 ratio across all samples. The sample was dried with a rotary vacuum concentrator and then dissolved in 30 µL 0.1% formic acid and 5% acetonitrile prior to mass spectrometric analysis.

### 2.7. Peptide Fractionation and LC-MS/MS Analysis

The TMT-labeled samples were analyzed using a LC-MS/MS system consisting of a DIONEX Ultimate 3000 HPLC system and a Q Exactive Plus (Thermo Fischer Scientific, France) equipped with an electrospray source.

Sample digests were analyzed with a capillary LC column (Thermo Fischer Scientific, Illkirch, France) PepMap100 C18, 300 μm × 15 cm, 2 μm particle, 100 Å pore size) and a 300 min gradient on the Q Exactive Plus (Thermo Fischer Scientific, Bremen, Germany). Sample digests were labeled with TMT 9-plex reagents at a ratio of 1:1 with a five-hour gradient, analyzed with a data dependent top 15 high-energy collision dissociation (HCD) method on the Q Exactive Plus. The resolution was 70K for full scan and 35K for MS2. The maximum injection time was 250 ms for both full scan and MS2 scan, top 15 HCD was selected with MS2 trigger threshold of 1E5 and dynamic exclusion of 60 s. Mobile phases A and B were composed of 0% and 100% acetonitrile, respectively, and each contained 0.1% formic acid. The LC gradient began with 2% B for 1 min and was ramped up to 8% B over 5 min, then to 35% B over 180 min, 55% B over 235 min, and finally to 90% B over 275 min. The column was re-equilibrated with 2% B for 15 min before the next run.

### 2.8. Protein Identification and Quantification

All MS raw data files were analyzed by Proteome Discoverer software 2.1.1.21 (Thermo Fisher, Illkirch, France) using the Sequest HT search engine against a database of *Mus musculus* protein sequences (Uniprot 10 090_Mus Musculus taxon 20 September 2018). X corr confidence was held upper to 0.7 for all parameters. Precursor mass tolerance was set to 10 ppm and fragment ion tolerance was 0.02 Da with permission of two missed cleavages in the trypsin digests. A decoy database search strategy was also used to estimate the false discovery rate (FDR) to ensure the reliability of the proteins identified: a 1% target FDR as strict criteria and 5% target FDR as relaxed criteria using Percolator. The strict maximum parsimony principle was performed, and only peptide spectra with at least low confidence were considered for protein grouping. Carbamidomethylation on cysteine (+57.021 Da) was set as static modifications. TMT6 tags on N termini (+229.163 Da) and on Lysine were set as static modifications. Two 10-plex technical replicates were acquired for two proteome experiments with the average and standard deviation reported.

For relative quantitation, both unique and razor peptides were considered a highly confident identification and used for quantification. Reporter ion abundances were corrected for isotopic impurities based on the manufacturer’s data sheets. Signal-to-noise (S/N) values were used to represent the reporter ion abundance with a co-isolation threshold of 75% and an average reporter S/N threshold of 10 and above required for quantitation spectra to be used. The S/N values of peptides, which were summed from the S/N values of the PSMs, were summed to represent the abundance of the proteins. The quantitative protein ratios were calculated and normalized by total peptide amount and scaled on channel average for each sample. For comparison, three identical biological control samples, labeled with TMT 126, 127N, and 127C, were used as references.

### 2.9. Bioinformatics Analysis

Pathway analyses were performed using Ingenuity Pathway analysis software (Qiagen, v.48207413, Courtaboeuf, France). Only pathways with *p*-values under a threshold of 0.01 were considered significant. Z-scores were also observed as a measure and a predictor for the activation state of the regulator. The activation Z-score makes predictions about potential regulators by using information about the direction of gene regulation. It can be used to infer the activation state of a putative regulator (i.e., whether the regulator is activated or inhibited). Protein networks were analyzed with STRING (10.5) (string-db.org) [31].

### 2.10. Statistical Analysis

Statistical analysis was performed using Prism (GraphPad software, San Diego, CA, USA). Dual comparisons were made using the Student’s t-test and comparisons between multiple conditions were analyzed using ANOVA (with Bonferroni’s post-test). Statistical significance was set at *p* < 0.05. The differentially expressed proteins (DEPs) were defined as proteins with a difference in expression above a factor of 1.3 and under 0.77 with a *p*-value below 0.05. Data are represented as mean ± S.D.

## 3. Results

### 3.1. Effect of ICM on Thyroid ^99m^pertechnetate Uptake in Humans

**Figure 1:**
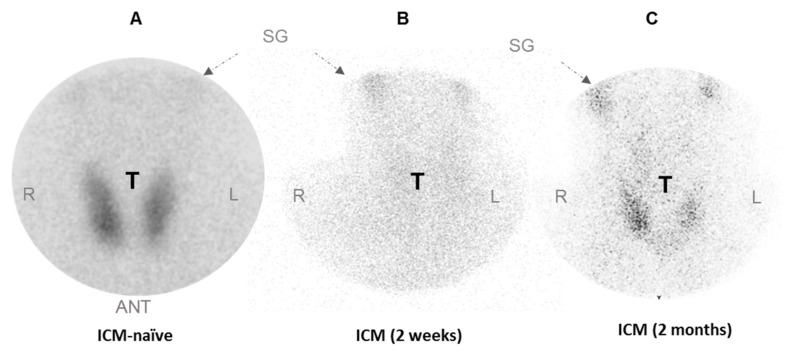
Effect of Iomeron on uptake of ^99m^TcO_4_^−^ by human thyroid. Scintiscans (where the pinhole collimator is centered on thyroids) of ICM-naïve patient (**A**) and patient given Iomeron 2 weeks (**B**) or 2 months before scintigraphy (**C**) are shown. ANT = anterior; SG = salivary glands; T = thyroid, L= left, R= right.

ICM has been shown to affect thyroid ^99m^TcO_4_^−^ uptake in humans [16]. To assess the duration of this effect, we measured ^99m^TcO_4_^−^ uptake in the thyroid of an ICM-naïve patient (Figure 1A) and a patient who had received an intravenous injection of Iomeron two weeks (Figure 1B) or two months before (Figure 1C). Figure 1 shows a scintiscan where the pinhole collimator is centered on the thyroid of an ICM-naïve patient in whom the thyroid was taking up ^99m^TcO_4_^−^. The scintiscan of a patient treated with Iomeron showed a lack of fixation in the thyroid region two weeks after ICM injection (Figure 1B). This uptake capacity remained low two months after a single administration of ICM (Figure 1C).

### 3.2. Effect of ICM and NaI on ^99m^pertechnetate Uptake in Mice

**Figure 2:**
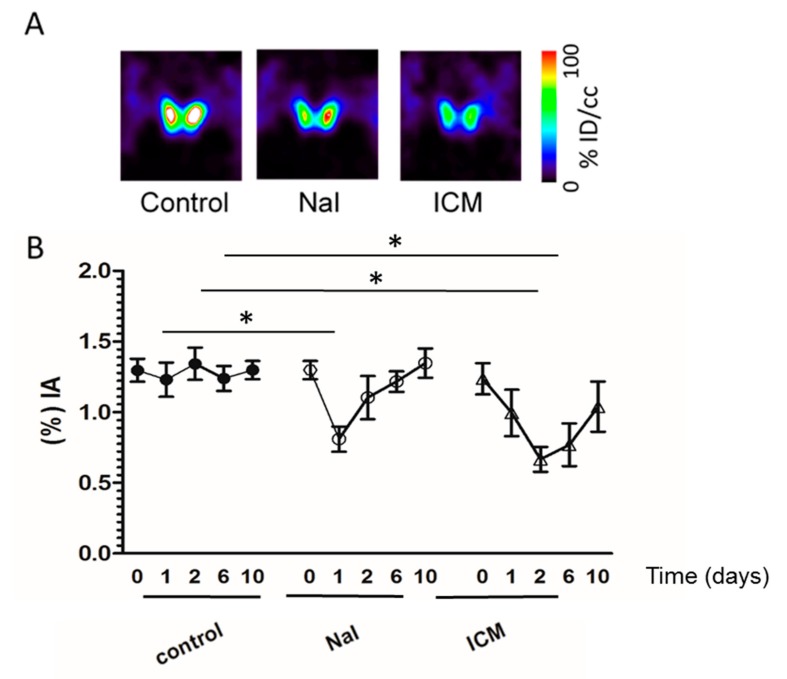
Effect of ICM and NaI on the uptake of ^99m^perctechnetate by the mouse thyroid. SPECT/CT imaging of mice administered 20 MBq ^99m^pertechnetate was performed (day 0). At the end of the scan, ICM or NaI was administered. One, two, six and ten days later, animals were injected with 20 MBq ^99m^pertechnetate and new scans were performed. (**A**) Representative microSPECT/CT images of thyroid uptake 24 hours after NaI treatment and 48 hours after ICM treatment. (**B**) The data represent the percentage of radiotracer injected taken up by the thyroid before treatment (0) and after one, two, six or ten days treatment with NaI (open circle) or ICM (open triangles). Uptakes in control thyroids are shown (filled circles). (n = 3 per condition). Significant differences (one-way ANOVA) in thyroid uptake are shown. Asterisks (* *p* < 0.05 versus control group) indicate results of post-hoc test (Bonferroni); % IA: percentage of the injected activity.

To further assess ICM inhibition in a quantitative manner, we next compared by SPECT/CT imaging the effect of NaI and iodinated contrast media (ICM) on the uptake of the iodide analog, ^99m^pertechnetate, by the mouse thyroid. Basal uptake was measured before and after administration of NaI and of ICM. Figure 2 shows that a marked reduction in radiotracer uptake by the thyroid was observed after both treatments. As shown on Figure 2B, this reduction reached up to 38% by 24 h after NaI administration (1.30 before treatment vs. 0.81 after NaI treatment, *p* = 0.03) and up to 50% by 48 h after ICM injection (1.30 vs. 0.65 after ICM treatment, *p* = 0.01).

### 3.3. Measurement of Iodide in Thyroids From Mice Treated with ICM or NaI

**Figure 3:**
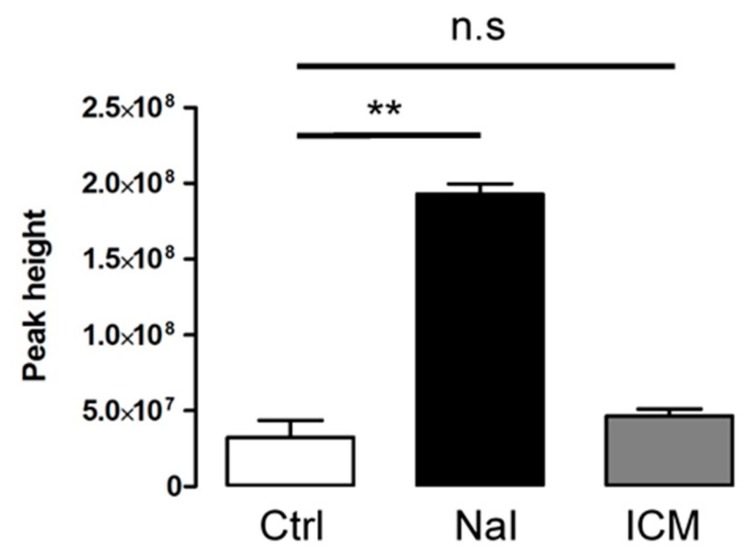
Assessment of iodide concentration through metabolomics comparison between ICM- and NaI-exposed thyroids. Samples for LC-MS measurement are analyzed after metabolites extraction from treated mouse thyroids (24 h after NaI, 48 h after ICM). Iodide concentration was evaluated by peak height from three independent organs. ** *p* < 0.01; n.s: non statistically significant.

We performed metabolomic analyses to compare the levels of iodide found in mouse thyroid upon administration of NaI or ICM. Figure 3 shows an elevated level of iodide in thyroids obtained from animals treated with NaI. By contrast, the level of iodide was not significantly affected in thyroids obtained from animals that received ICM up to 48 h after treatment. These results emphasize the fact that ICM affects thyrocyte function independently of free-iodide.

### 3.4. Quantitative Proteomic Analysis of Thyroids From Mice Treated With ICM or NaI

**Figure 4:**
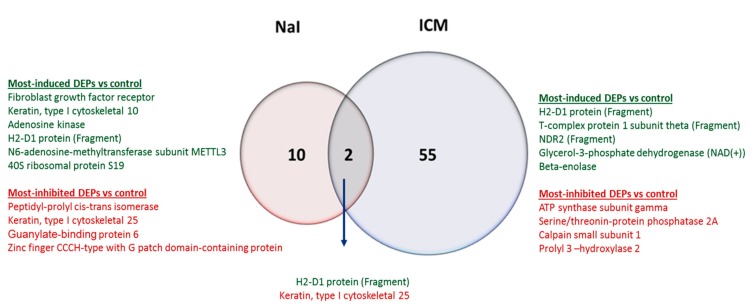
Identification of the significant differentially expressed proteins (DEPs) induced by NaI and ICM treatments. A Venn diagram illustrating the common and unique DEPs in the thyroid between NaI and ICM. The numbers represent the proportion of significantly changed proteins in each comparison with control thyroids.

We next analyzed thyroid proteome upon either NaI or ICM administration and compared it to the proteome of thyroid of control animals. As the maximal radiotracer uptake reduction is observed at 24 h following NaI injection (using either ^99m^Tc or ^123^I [16], as radiotracers), and 48 h after ICM treatment, 24 h (for NaI) and 48 h (for ICM) were chosen to assess the changes in the proteome associated with the inhibitory effects on mouse thyroids. The results are presented as ratios of differentially expressed proteins (DEPs) between NaI and control or between ICM and control. The DEPs were defined as proteins with a difference in expression above a factor of 1.3 and under 0.77 with a *p*-value below 0.05 (Table 1 and Table 2). A total of 12 and 57 DEPs were identified in NaI versus control and ICM versus control, respectively (Figure 4). From these DEPs, only two proteins are commonly differentially expressed in NaI versus control and in ICM versus controls thyroids. These proteins are H2-D1 (Q3KQJ3) and Krt25 (Q8VCW2). H2-D1 is the most induced in the ICM set. The most induced and most inhibited DEPs in NaI versus control are listed in Table 1 and those in ICM versus control are shown in Table 2, and on Figure 4. An enriched list of proteins was established that contains a total of 72 proteins and 148 proteins identified with a *p*-value under 0.05 in NaI versus control and in ICM versus control, respectively (Supplementary Tables S1 and S2).

### 3.5. Altered Pathways

**Figure 5:**
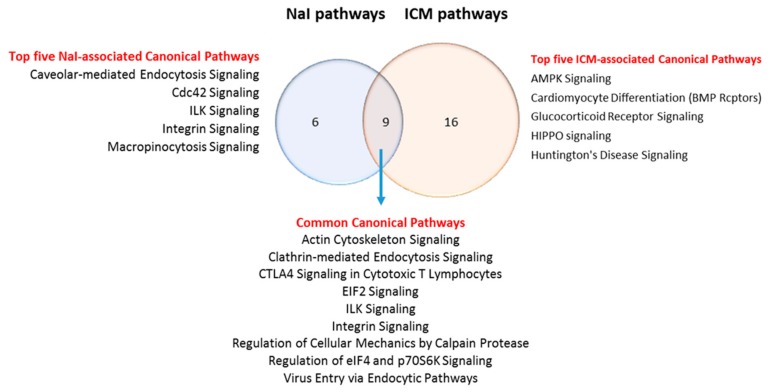
The DEPs in both treatments show up in different networks, which lead to different pathways. IPA analysis of the altered pathways shows that ICM treatment leads to 25 pathways while NaI leads to 15 pathways. The Venn diagram illustrates six exclusive pathways in the NaI group versus 16 exclusive pathways analysis in ICM group, with nine pathways being common to both treatments

To determine the top pathways altered by NaI or ICM, the whole DEPs dataset was analyzed using Ingenuity Pathway analysis (IPA) with the enriched list of proteins (Figure 5). Statistically significant canonical pathways for which the *p*-values < 0.01 were identified. Nine pathways are common to the two enriched datasets (Table 3). They include metabolic pathways and signaling pathways. Only six are exclusive to the NaI versus control enriched dataset (Supplementary Table S3). Finally, 16 pathways are exclusive to the ICM versus control enriched dataset (Supplementary Table S4). Networks of DEPs were created using STRING (Figure 5). A higher density of interactions can be observed in the DEP/ICM versus control dataset than in the DEP/NaI versus control dataset.

### 3.6. Commonly Modulated Pathways

**Figure 6:**
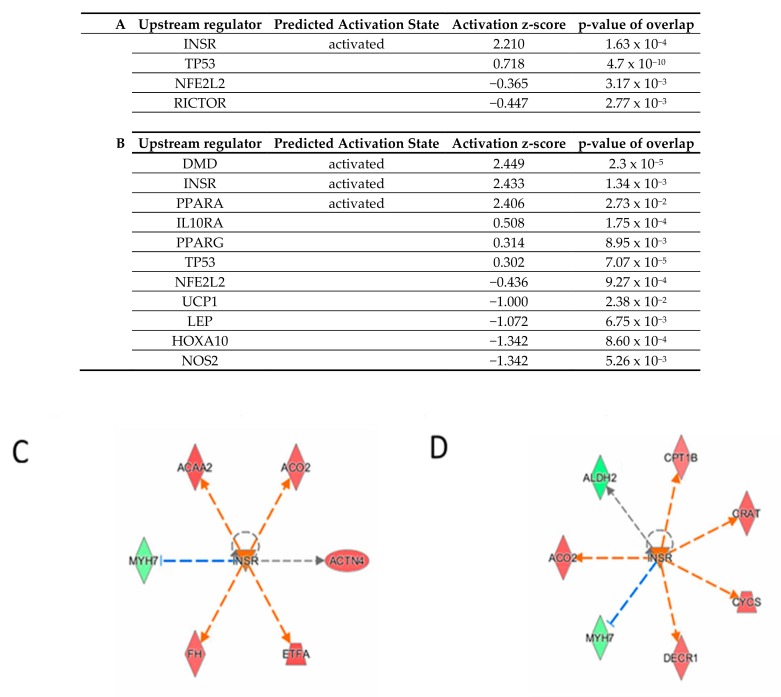
Upstream analysis of DEPs from NaI vs. CTRL set (**A**) and ICM vs. CTRL set (**B**). Activated (z-score ≥ 2) and inhibited (z-score ≤ −2) upstream regulators are highlighted in orange and blue, respectively. (**C**,**D**) INSR regulated proteins in NaI and ICM treatments. Up-regulated and down-regulated proteins are highlighted in red and green, respectively, and the color depth is correlated to the fold change. Orange and blue dashed lines with arrows indicate indirect activation and inhibition, respectively. Gray dashed lines with arrows depict no prediction.

To look for potential common regulation mechanisms in accordance with common reduced NIS expression and reduced iodide uptake, the upstream analysis module of Ingenuity Pathway Analysis was run on the nine total pathways commonly modulated in the two conditions (ICM and NaI). Insulin Receptor (INSR) was identified as a significantly unbiased upstream activator in both treatments: NaI (z-score = 2.219, *p* = 1.63 × 10^−4^) and ICM (z-score = 2.433, *p* = 1.34 × 10^−4^) (Figure 6) and predictions of activated and inhibited candidates in each treatment are shown (Figure 6C,D).

### 3.7. Focus on the TSH Receptor

**Figure 7:**
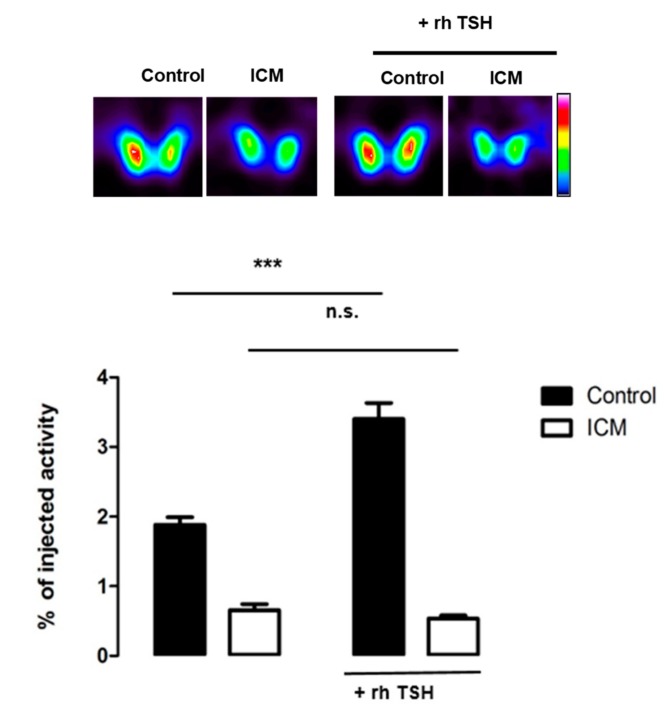
^99m^ Pertechnetate thyroid uptake on control and ICM-treated animals before and after recombinant human thyroid-stimulating hormone (TSH) administration. SPECT/CT imaging on control and ICM-treated mice was performed before and 24 h after intraperitoneal administration of 3.2 μg/mouse of TSH. The data represent the averaged values of radiotracer accumulation in the thyroid at 60 min (n = 4 per group). ns: not statistically significant; ***: *p* < 0.001.

Considering its pivotal role in thyroid function, we decided to focus on the regulation of the TSH receptor in ICM-treated animals. As this protein is not abundant, we had to enlarge the overview of the proteome by increasing the false discovery rate to 15%. The other parameters of the analysis were kept unchanged. The TSH receptor was found to be downregulated in thyroids from ICM-treated animals versus control (fold change 0.68, *p*-value = 4 × 10^−3^). To determine whether this decrease had physiologically-relevant implications, we examined whether thyroids from control or ICM-treated animals were responsive to TSH. Figure 7 shows that administration of human, recombinant TSH into control mice leads to an increase in 99-m pertechnetate uptake (used as a surrogate for I-uptake, [5]) by the thyroid. In mice treated with ICM, radiotracer uptake was lower one day after ICM administration, and administration of human recombinant TSH failed to increase radiotracer uptake significantly (Figure 7).

## 4. Discussion

In a recent report, we provided an indirect but strong argumentation based on SPECT/CT imaging of ^123^I and ^99m^Tc to demonstrate that ICM reduce thyroid uptake of iodide independently of free iodide [16]. The effect of ICM is thyroid-specific, not exerted directly on isolated thyrocytes in vitro, and involves a dramatic decrease in NIS expression in thyrocytes [16]. In patients, thyroid uptake capacity can remain low two months after a single administration of ICM. This effect is not consistent with a transient Wolff–Chaikoff effect induced by free iodide, as shown in our experimental imaging data. In the present study, measurement of total iodide in the thyroid upon administration of NaI showed, as expected, a marked increase in free-iodide in the thyroid. This is likely to be due to the large amount of iodide entering NIS-expressing thyrocytes. This entry results in intracellular events leading to a classical Wolff–Chaikoff effect for which a consequence is a reduction in the expression of the NIS protein [2]. Measurement of the iodide levels in the thyroids of animals treated with ICM failed to show a significant difference up to 48 h after treatment. Our observation in mice is in agreement with a dataset obtained in humans in which the authors demonstrated that intra-thyroidal iodine was unaffected by ICM (up to 96 h following ICM administration) [32]. Together with our previous study showing that ICM (and its contaminating free iodide) fails to directly affect thyrocytes uptake in vitro, the present data confirm that ICM induces thyroid stunning independently of free-iodide.

To get further insight into the mechanisms involved in thyroid uptake inhibition, we used a quantitative proteomic approach to examine the variations in protein content in thyroids exhibiting maximal radiotracer uptake reduction after either NaI or ICM treatment. Overall, our data demonstrate that ICM induces a dramatic change in the level of many different proteins affecting numerous pathways. In comparison to ICM, administration of NaI leads to a lower level of changes. Only 10 proteins are differentially expressed in thyroids from NaI-treated animals in contrast to 55 in ICM-exposed thyroids, with two proteins being commonly differentially expressed. When pathways are considered, the similarity between the two conditions is much higher: about 30% of total pathways are commonly modulated in the two conditions and these modulated pathways could be the signature of common mechanisms leading to reduced NIS expression and reduced iodide uptake. One of these pathways is the regulation of the eIF4 and p70S6K signaling pathway. The upstream analysis module of Ingenuity Pathway Analysis suggested that the insulin receptor (INSR) was a significantly unbiased upstream activator in both treatments. INSR upregulates the mammalian target of rapamycin (mTOR), which is known to downregulate iodide uptake in thyrocytes [33]. mTOR is regulated by phosphoinositide 3-kinase/Akt (PI3K/Akt), which has been involved in the acute inhibitory effect of iodide excess on the Na/I symporter [34], and is generally recognized as important in NIS regulation [35,36,37]. It could also be involved in the inhibitory effect exerted by ICM. The TCA cycle is unregulated uniquely in NaI-administrated mice.

In addition to these common effects, our proteomic analysis reveals that ICM induce a dramatic change in cellular proteins leading to the modulation of 16 distinct pathways. This profound modification of the thyroid state could be responsible for the long-lasting effects of ICM.

The effect of ICM on TSH-induced iodide uptake by the thyroid is not well-documented. A clinical study reported that the inhibition of iodide uptake by the thyroid could be reversed by intramuscular administration of recombinant human TSH [38]. However, the ICM stunning reported in this study was relatively low and a large heterogeneity was observed in the nine patients of the cohort [38]. In another study, the anecdotic case of a patient with differentiated thyroid carcinoma and who had previously received ICM was reported. This patient showed a lack of TSH-induced iodide uptake at the site of tumor recurrence or thyroid remnants [39]. Isobaric chemical labelling of peptides provides a reliable quantitative method to compare protein expression in different conditions [25,26]. However, this accuracy is obtained at the expense of sensitivity and lowly expressed proteins are not necessarily detected. The TSH receptor can be detected if the false discovery rate is increased to 15%. This issue can be overcome by protein fractionation upstream of proteomic analysis; this fractionation can be based on isoelectric point or subcellular compartment, for example. Our functional data show unambiguously that administration of ICM reduces TSH receptor expression which results in a loss of TSH-induced iodide uptake by the thyroid. These data, predicted by the proteomic analysis, demonstrate that the cellular changes induced by ICM affect the TSH receptor in addition to NIS [16].

In the present study, however, we failed to detect NIS proteins and therefore to analyze protein post-translational modifications. In the literature, several key protein modifications (phosphorylation, glycosylation, ubiquitination of tyrosine residues, etc.) have been described by different groups including ours to critically affect NIS interactions with intracellular partners, its subsequent proper localization on the plasma membrane, as well as its activity [40,41,42,43]. Although beyond the scope of the present work, a longitudinal study addressing at once the transcriptional, translational, and post-translational levels of NIS regulation in wild-type mice subjected to NaI versus ICM could improve the comprehension of ICM effect. Additional findings on the fine regulation of NIS may have consequences of paramount importance in the clinical setting where low expression of membranous NIS exists, not only to limit iodine refractoriness and avoid related morbidity, but also to enable earlier treatment with small molecule inhibitors [42,44,45,46,47,48,49,50].

Guidelines recommend a delay in the radioactive-iodide treatment of patients with differentiated thyroid carcinoma who have been injected previously with ICM [9,11,12,13]. As upon ICM injection urinary iodide levels are elevated for several weeks [19,20,21,22] and as free-iodide was considered to be the cause of thyroid stunning by ICM [9,38], urinary iodide content has been proposed as a surrogate marker of recovery of iodide uptake by the thyroid [12,19,20,21,22]. In the light of our results, the relevance of the measurement of urinary iodide to predict the recovery of iodide uptake by the thyroid is questionable. To determine in patients the duration of the decrease in NIS functional expression with the aim of refining the currently recommended four-to-six weeks before a radioiodine therapy, we advocate for the need of a dedicated trial. Although this problem is highly relevant for cancer patients, it is difficult to envisage a trial involving this category of individuals. One possibility could be to have a group of healthy subjects who would receive intravenous administration of ICM and a control group. A scintigraphy aiming at measuring the uptake of iodide by the thyroid would be performed at time zero and then a group would receive an intravenous administration of the ICM while the other would receive a mock injection. This scintigraphy would be repeated on a weekly basis until the radiotracer uptake in the ICM group returns to the value obtained before the administration of the contrast agent. In addition, the measurement of urinary iodide could be performed to determine whether there is a correlation between these parameters and the capacity of the thyroid to take up iodide.

Numerous volatile radionuclides are emitted as a result of a nuclear incident, amongst which 131-I is abundant. The well-established preventive countermeasure to protect populations is the ingestion of potassium iodide (KI) tablets that prevent the entry of 131-I to the thyroid. Nevertheless, the effect of KI tablets is transient, and the protective effect only lasts for 24 h [51,52]. As a result, the United States Food and Drug Administration recommends KI tablets to be ingested on a daily basis in cases of prolonged 131-I exposure [53] such as those observed in the Fukushima prefecture [54]. Recently, we have provided the evidence that the sublingual delivery of ICM is efficient in mice and could be considered in humans [16]. In addition, randomized, multicenter evaluations of the safety profile of iodinated contrast agents are widely available [55,56,57,58,59,60,61,62,63]. Considering the safety profile of ICM, the long-lasting effect of single ICM sublingual administration on thyroid iodide uptake and in the light of the mechanistic observations presented in this report, we advocate that formulations based on ICM could be used in conjunction or instead of KI tablets in radioprotection. In that regard, dedicated studies will be needed.

In conclusion, our study demonstrates that exposure of thyroid to NaI modulates 15 cellular pathways. Most of these pathways (9/15) are also affected by exposure of thyroids to ICM. This observation suggests that common intracellular mechanisms are involved in the reduction of iodide uptake promoted by the two effectors. However, the fact that ICM fails to affect thyroid iodide content suggests that the modulation of these common pathway is triggered by separate effectors. In addition, ICM modulate 16 distinct pathways and these large and dramatic cellular changes may be responsible for the long-lasting effect of ICM on iodide uptake by the thyroid. These observations may have implication in the management of patients affected by differentiated thyroid carcinomas who have been exposed to ICM. They also provide the basis for the utilization of ICM-based compounds in radioprotection of the thyroid.

## Figures and Tables

**Table 1:** Top 10 list of the most induced and most inhibited proteins in NaI vs. CTRL.

Accession	Description	Gene	Abundance Ratio NaI vs. CTRL	*p*-Value
A1YYN9	Fibroblast growth factor receptor	Fgfr2	1.44	0.02
P02535	Keratin, type I cytoskeletal 10	Krt10	1.32	0.01
P55264	Adenosine kinase	Adk	1.32	0.05
Q3KQJ3	H2-D1 protein (Fragment)	H2-D1	1.31	0.02
G3UXX7	N6-adenosine-methyltransferase subunit METTL3 (Fragment)	Mettl3	1.30	0.04
D3Z722	40S ribosomal protein S19	Rps19	1.29	0.01
Q64378	Peptidyl-prolyl cis-trans isomerase FKBP5	Fkbp5	0.59	0.02
Q8VCW2	Keratin, type I cytoskeletal 25	Krt25	0.51	0.04
A0A0G2JDV3	Guanylate-binding protein 6	Gbp6	0.49	0.01
F7C2Y9	Zinc finger CCCH-type with G patch domain-containing protein (Fragment)	Zgpat	0.35	0.01

**Table 2:** Top 10 list of the most induced and most inhibited proteins in ICM vs. CTRL.

Accession	Description	Gene	Abundance Ratio ICM vs. CTRL	*p*-Value
Q3KQJ3	H2-D1 protein (Fragment)	H2-D1	1.75	0.05
H3BJB6	T-complex protein 1 subunit theta (Fragment)	Cct8	1.73	0.03
Q8CGR9	NDR2 (Fragment)	Ndr2	1.47	0.04
P13707	Glycerol-3-phosphate dehydrogenase [NAD(+)], cytoplasmic	Gpd1	1.46	0.04
P21550	Beta-enolase	Eno3	1.46	0.04
Q3UD06	ATP synthase subunit gamma	Atp5c1	0.48	0.05
G3UWS4	Serine/threonine-protein phosphatase 2A 65 kDa regulatory subunit A beta isoform	Ppp2r1b	0.45	0.03
O88456	Calpain small subunit 1	Capns1	0.43	0.02
Q8CG71	Prolyl 3-hydroxylase 2	P3h2	0.37	0.03
Q3UHW5	Uncharacterized protein	Rab8a	0.33	0.03

**Table 3:** Common pathways list affected in NaI-treated animals and ICM-treated animals.

	-LOG(*p* Value)			Z Score				
Canonical Pathway	(NaI) vs. (CTRL)	(ICA) vs. (CTRL)	(ICA) vs. (NaI)	(NaI) vs. (CTRL)	(ICA) vs. (CTRL)	(ICA) vs. (NaI)	NaI Proteins	ICA Proteins
Actin Cytoskeleton Signaling	4.291	2.503	1.423	1	0.377	N/A	ITGB1,FLNA,FGFR2,MYLK,MYH7,ACTN4,NCKAP1	ITGB1,ACTR3,PTPN11,Actn3,MYH7,GSN,ACTG1
Clathrin-mediated Endocytosis Signaling	4.695	2.860	1.583	N/A	N/A	N/A	ITGB1,PICALM,FGFR2,SERPINA1,UBC,CTTN,AP1G1	ITGB1,HSPA8,ACTR3,PTPN11,UBC,ACTG1,AP1G1
CTLA4 Signaling in Cytotoxic T Lymphocytes	2.140	2.113	0.583	N/A	N/A	N/A	HLA-A,FGFR2,AP1G1	PTPN11,HLA-A,PPP2R1B,AP1G1
EIF2 Signaling	2.718	3.432	0.835	N/A	N/A	N/A	RPL27,PABPC1,RPS19,EIF5,FGFR2	PABPC1,RPS7,PTBP1,EIF3B,PTPN11,EIF2S3,EIF3E,RPS3
ILK Signaling	3.785	2.250	0.331	0	0.816	N/A	ITGB1,FLNA,FERMT2,FGFR2,MYH7,ACTN4	ITGB1,PTPN11,Actn3,MYH7,PPP2R1B,ACTG1
Integrin Signaling	3.537	3.359	1.497	2.449	1.133	N/A	ITGB1,FGFR2,CAPN2,MYLK,ACTN4,CTTN	ITGB1,CAPNS1,ACTR3,PTPN11,CAPN1,Actn3,GSN,ACTG1
Regulation of Cellular Mechanics by Calpain Protease	2.593	2.692	0.725	N/A	N/A	N/A	ITGB1,CAPN2,ACTN4	ITGB1,CAPNS1,CAPN1,Actn3
Regulation of eIF4 and p70S6K Signaling	2.381	5.238	0.404	N/A	N/A	N/A	PABPC1,ITGB1,RPS19,FGFR2	PABPC1,RPS7,ITGB1,EIF3B,PTPN11,EIF2S3,EIF3E,RPS3,PPP2R1B
Virus Entry via Endocytic Pathways	3.907	2.563	1.276	N/A	N/A	N/A	ITGB1,FLNA,HLA-A,FGFR2,AP1G1	ITGB1,PTPN11,HLA-A,ACTG1,AP1G1

## Supplementary Materials

The following are available online at https://www.mdpi.com/2077-0383/9/2/329/s1, Table S1: Enriched list of the most induced and most inhibited proteins in NaI vs. control thyroids. Table S2: Enriched list of the most induced and most inhibited proteins in ICM vs. control thyroids. Table S3: Enriched list of the 15 pathways exclusive to the NaI versus control thyroids. Table 4 Enriched list of the 25 pathways exclusive to the ICM versus control thyroids.
